# Editorial Comment: Penile revascularization utilizing the lateral femoral circumflex artery after pelvic fracture urethral injury

**DOI:** 10.1590/S1677-5538.IBJU.2023.05.02

**Published:** 2023-06-25

**Authors:** Luciano A. Favorito

**Affiliations:** 1 Universidade do Estado do Rio de Janeiro Unidade de Pesquisa Urogenital Rio de Janeiro RJ Brasil Unidade de Pesquisa Urogenital - Universidade do Estado do Rio de Janeiro - Uerj, Rio de Janeiro, RJ, Brasil

Koch G 1, Gleicher S 1, Criman E 1, Sandvall B 1, Johnsen N 1

**J Sex Med. 2023; 20:(suppl1), qdad060.412.**

**DOI: 10.1093/jsxmed/qdad060.412 | ACCESS: qdad060.412**

## COMMENT

The present paper shows how the anatomy is important to urology. The authors demonstrate the use of an alternative arterial donor vessel for penile revascularization.

Using an operating microscope the dorsal penile artery was ligated and the distal end was anastomosed to the donor artery and an end-to-end fashion with the lateral femoral circumflex artery. Penile vascular anatomy is basic for this procedure. The penis is irrigated by two internal pudendal arteries, branches of the internal iliac (hypogastric) artery. After its various perineal branches, the pudendal arteries combine to form the so-called common penile artery, which divides into three branches: the bulbourethral artery, the dorsal penile artery and the cavernosal artery. The cavernosal artery is located inside the corpus cavernosum, the bulbourethral artery is responsible for irrigating the corpus spongiosum and urethra, and the dorsal penile artery is located between the tunica albuginea and Buck’s fascia (1, 2).

The authors concluded that this case demonstrates an excellent result from penile revascularization utilizing the lateral femoral circumflex artery. This artery can be considered an alternate donor for penile revascularization procedures following pelvic fracture urethral injury when there is a contraindication to the use of the inferior epigastric artery.
